# Timothy mutation affects tightly sealing point of Ca_V_1.2 activation gate

**DOI:** 10.1186/1471-2210-11-S2-A12

**Published:** 2011-09-05

**Authors:** Katrin Depil, Stanislav Beyl, Anna Stary-Weinzinger, Annette Hohaus, Eugen Timin, Steffen Hering

**Affiliations:** 1 Institute of Pharmacology and Toxicology, University of Vienna, 1090 Vienna, Austria

## Background

The Timothy syndrome (TS) mutations G402S and G406R abolish inactivation of Ca_V_1.2 and cause multiorgan dysfunction and lethal arrhythmias.

## Methods

In order to gain insights into the consequences of the G402S mutation on structure and function of the channel, we systematically mutated the corresponding G432 and the homologous S6 positions of the other three domains of the rabbit channel and applied homology modeling.

## Results

Homology modeling revealed that G432 forms part of a highly conserved structure motif (G/A/G/A) of small residues in homologous positions of all four domains (G432 (IS6), A780 (IIS6), G1193 (IIIS6), A1503 (IVS6)). In contrast, corresponding mutations in domains II, III and IV induced parallel shifts of activation and inactivation curves indicating a preserved coupling between both processes. Disruption between coupling of activation and inactivation was specific for mutations of G432 in domain I. Mutations of G432 removed inactivation irrespective of the changes in activation. In all four domains residues G/A/G/A are in close contact with larger bulky amino acids from neighboring S6 helices.

## Conclusions

These interactions apparently provide adhesion points thereby tightly sealing the activation gate of Ca_V_1.2 in the closed state. Such a structural hypothesis is supported by changes in activation gating induced by mutations of the G/A/G/A residues.

